# Factors related to the burden of family caregivers of elderly patients with spinal Tumours in Northwest China

**DOI:** 10.1186/s12883-020-01652-0

**Published:** 2020-02-28

**Authors:** Jing Luo, Yongchun Zhou, Haiping Liu, Jing Hu

**Affiliations:** 1 Department of Nursing Administration, Honghui Hospital, Xi’an Jiaotong University College of Medicine, 555# You-yi East Road, Xi’an, 710054 Shaanxi People’s Republic of China; 2 Department of Orthopedics, Shaanxi Provincial People’s Hospital, Xi’an, Shaanxi People’s Republic of China; 3 Department of Spine Surgery, Honghui Hospital, Xi’an Jiaotong University College of Medicine, Xi’an, People’s Republic of China

**Keywords:** Spinal tumour, Family caregivers, Burden, Factors

## Abstract

**Background:**

Family caregivers of elderly patients with spinal tumours experience considerable pain and burden during the care process. This study aims to investigate the factors associated with caregiver burden in family caregivers of elderly patients with spinal tumours.

**Methods:**

A total of 220 elderly patients with spinal tumours (age ≥ 65 years) hospitalized at the spine centre of our hospital from January 2015 to December 2017 and their family caregivers were recruited for this cross-sectional study. All participants completed a sociodemographic questionnaire. Caregiver burden, social support and self-efficacy were assessed by the Chinese version of the Zarit Burden Interview (ZBI), the Social Support Rating Scale (SSRS) and the General Self-Efficacy Scale (GSE), respectively. The factors related to caregiver burden were analysed by multivariate analysis. *P* < 0.05 was considered statistically significant.

**Results:**

The 216 elderly patients with spinal tumours were 71.59 ± 8.49 years old, and their caregivers were 70.46 ± 9.13 years old. A total of 170 patients were cared for by their spouses, who accounted for 78.7% of all caregivers. The ZBI score for the family caregivers was 35.5 ± 7.5, and most caregivers (84.5%) reported a moderate or heavy burden. The factors related to caregiver burden included patient paralysis, the primary cancer site, chemotherapy and/or radiation, cognitive dysfunction, functional status, monthly income, pain score, caregivers’ SSRS score, and GSE score.

**Conclusions:**

Most family caregivers of elderly patients with spinal tumours have a considerable caregiver burden. Interventions based on social support and self-efficacy can help reduce caregiver burden.

## Background

‘Chronic diseases (e.g., tumours) represent a central event that constitutes a major challenge for the family. These diseases have physical, psychological, socioeconomic, and behavioural effects on patients and their family caregivers that translate into vulnerability as well as decreased quality of life and family functioning’ [1]. The spine is a common site of bone metastasis for malignant tumours, accounting for 5–10% of all bone metastases [2, 3]. With the ageing of the population and the increase in the average life expectancy, the incidence of spinal tumours in the elderly has increased significantly in recent years, and spinal tumours have become one of the major chronic diseases in China. With their associated high mortality, disability, medical risk and medical cost, these tumours seriously affect the physical and mental health of the elderly. Surgical treatment can significantly alleviate pain, rebuild spinal stability and relieve spinal cord compression [4, 5]. The management of malignant spinal tumours is complex. Care for patients with malignant spinal tumours requires considerable resources, including long-term medical services and caregiver support.

In China, especially in Northwest China, due to limited public medical resources, many patients with spinal tumours need to be cared for at home. In addition, there is a tradition of family rehabilitation in China, so family caregivers are usually patients’ primary caregivers. In the literature, ‘a family caregiver is defined as a person who has a significant emotional bond with the patient; this caregiver is a family member who is a part of the patient’s family life cycle; offers emotional-expressive, instrumental, and tangible support; and provides assistance and comprehensive care during the chronic illness, acute illness, or disability of a child, adult, or elderly person’ [6]. Family caregivers play a key role in the management of patients with spinal tumours and often experience tremendous pressure and caregiving burden. Patients with spinal tumours and their caregivers are also under psychological and emotional stress. ‘Resilience to disease is a process of positive adaptation despite the loss of health; it involves the development of vitality and skills to overcome the negative effects of adversity, risks, and vulnerability caused by disease’ [7]. Previous studies have shown that the family caregivers of patients with spinal tumours often have poor quality of life and face immense psychological and economic stress [8, 9]. Our previous study showed that caregivers’ monthly income can increase family caregivers’ burden [10]. In China, little information is available on the factors related to caregiver burden for patients with spinal tumours. Regarding other factors, such as the patient’s paralysis, pain score, primary cancer, chemotherapy or (and) radiation, cognitive dysfunction, functional status, GSE score, and SSRS score; the caregiver’s residence, care time, age, gender, education level and relationship to the patient; the patient’s age, gender, education level and tumour site, it is unknown whether these are positively correlated with caregiver burden. In addition, protective psychosocial factors (social support and self-efficacy) whether influence and decrease to the Caregivers burden remains unknown, too.

Understanding the specific factors of caregiver burden is important for helping family caregivers cope with their burden and can help hospitals and governments improve the current situation of care for patients with spinal tumours. Moreover, because of the correlation of caregiver burden with the well-being of caregivers, it is necessary to address caregiver burden so that health care professionals can meet the needs of family caregivers. Therefore, this study aims to investigate the factors related to the burden of family caregivers of elderly patients with spinal tumours and to assess the demographic and clinical characteristics of these patients and their family caregivers.

## Methods

### Study participants

A total of 220 family caregivers of elderly patients (age, ≥ 65 years) with spinal tumours (primary or metastatic) undergoing surgery were investigated in Northwest China. This instrument-based empirical study was conducted using a cross-sectional nonexperimental design and intentional nonprobability sampling. Spinal tumours were diagnosed based on clinical manifestations, imaging examinations (X-ray, CT, MRI), and postoperative pathological examination. The other inclusion criteria were that the family caregivers must be patients’ family members and must be between 20 and 75 years old. The exclusion criteria were as follows: 1) had mental illness, 2) did not sign the informed consent form, 3) was not able to understand the questions, 4) had been a caregiver for less than 2 months, or 5) refused or withdrew from participation. The Ethics Committee of Honghui Hospital, Xi’an Jiaotong University College of Medicine, approved the study protocol. All patients and caregivers provided written informed consent agreeing to the use and publication of their data for research purposes.

### Data collection

Data collection was performed by trained personnel at Honghui Hospital, Xi’an Jiaotong University College of Medicine, under the direction of the first author. The data collection process lasted approximately 36 months, from January 2015 to December 2017. The patients and their family caregivers completed a sociodemographic questionnaire. The demographic statistics collected from the patients included their age, gender and education level. The demographic statistics collected from the family caregivers included their age, gender, education level, place of residence, relationship with the patient, duration of care and monthly income per person. The patients’ clinical diagnosis, including the spinal tumour site, primary cancer site, cognitive dysfunction level, functional status, chemotherapy and/or radiation treatment, paraplegia and degree of pain, was obtained from medical records. After the objectives, methods, benefits and potential risks of this study were explained, consenting caregivers signed the informed consent form and participated in face-to-face interviews [11]. Data were collected in the patients’ homes, and the patients were not allowed to be present during the interviews to ensure confidentiality.

### Evaluation standard

Caregivers were also asked to complete three tools to determine their burden, social support and self-efficacy.

Caregiver burden in this study was assessed by means of the 22-item self-report Zarit Burden Interview (ZBI) [12]. The Chinese version of the ZBI is reliable and effective for Chinese caregivers [13]. A five-point Likert-type scale ranging from 0 to 4 was applied to score responses. For items 1–21, respondents indicated their endorsement of each statement (0-never; 1-rarely; 2-sometimes, 3-quite frequently, and 4-nearly always). For the last item, respondents rated how overwhelmed they felt as a caregiver (0-not at all; 1-a little, 2-moderately, 3-quite a bit, and 4-extremely). A higher score indicates a greater perceived burden. The scale’s overall internal consistency is very high (Cronbach’s alpha = 0.88) [13].

Social support was evaluated by the Social Support Rating Scale (SSRS) [14]. This is a 3-subscale measure with 10 items: 3 items for evaluating objective support, 4 for evaluating subjective support, and 3 for evaluating social support. High scores indicate a high level of social support. The scale’s overall internal consistency is very high (Cronbach’s alpha = 0.90) [14].

The self-efficacy of caregivers was measured using the 10-item version of the General Self-Efficacy Scale (GSE) [15]. A four-item response scale was used that ranged from “completely wrong” to “completely right.” High scores indicate a high level of self-efficacy. The scale’s overall internal consistency is very high (Cronbach’s alpha = 0.91) [15].

### Statistical analysis

Data were statistically processed with SPSS 19.0 (SPSS, IL, USA). The measurement data were expressed as “x ± s” and analysed by t-test. The enumeration data were expressed as percentages (%). The differences between two groups were analysed by t-test and among multiple groups by analysis of variance. Correlations were calculated using Spearman’s rank correlation coefficient. Subsequently, the important factors in the univariate correlation analysis were included in a multivariate linear stepwise regression, in which caregiver burden was used as a dependent variable. For all tests, *P* < 0.05 was considered statistically significant.

## Results

A total of 220 caregivers were recruited in the study, 4 of whom were excluded: 2 refused to participate and 2 dropped out halfway. The final sample size was 216 (response rate, 98.18%).

### Characteristics of the patients

The 216 patients with spinal tumours were 71.59 ± 8.49 years old, and 53.3% were female. Among the patients, 67.1% had received a primary education or less, 34.7% had paralysis and 78.7% had moderate to severe pain. Twenty-seven percent of patients had primary spinal tumours, 48.6% had tumours in the thoracic spine, and 24.5% received radiation and chemotherapy (Table 1).

**Table 1:** Demographic and clinical characteristics of the patients and their caregivers

Variable	Patients (n = 216)	Caregivers (*n* = 216)
Age (years)	71.59 ± 8.49	70.46 ± 9.13
Gender		
Female	115 (53.3%)	90 (41.7%)
Male	101 (46.7%)	126 (58.3%)
Education		
≦Primary	145 (67.1%)	152 (70.4%)
≧Secondary	71 (32.9%)	64 (29.6%)
Place of residence		
Rural	N/A	131 (60.6%)
Urban	N/A	85 (39.4%)
Relationship		
Spouse	N/A	170 (78.7%)
Other	N/A	46 (21.3%)
Duration of caregiving		
*<* 6 months	N/A	150 (69.4%)
> 6 months	N/A	66 (30.6%)
Monthly income		
*<* 200 US	N/A	36 (16.7%)
200–500 US	N/A	125 (57.8%)
> 500 US	N/A	55 (25.5%)
Primary cancer site		
Lung	36 (16.7%)	N/A
Gastrointestinal tract	38 (17.6%)	N/A
Breast	34 (15.7%)	N/A
Other (bladder, prostate, cervix, colon)	49 (22.7%)	N/A
Spine	59 (27.3%)	N/A
Chemotherapy and/or radiation for tumour		
Chemotherapy	105 (48.7%)	N/A
Radiation	58 (26.8%)	N/A
Chemotherapy and radiation	53 (24.5%)	N/A
Cognitive dysfunction [16]		
Memory	11 (5.1%)	N/A
Depression	42 (19.4%)	N/A
Disruptive behaviour	9 (4.2%)	N/A
None	154 (71.3%)	N/A
Functional status (activities of daily living) [17]		
Fully independent	65 (30.1%)	N/A
Fully dependent	52 (24.1%)	N/A
Other	99 (45.8%)	N/A
Spinal tumour site		
Cervical	34 (15.8%)	N/A
Thoracic	105 (48.6%)	N/A
Lumbar	77 (35.6%)	N/A
Paralysis		
Yes	75 (34.7%)	N/A
No	141 (65.3%)	N/A
Pain, VAS score		
≦3	46 (21.3%)	N/A
4–6	105 (48.6%)	N/A
≧7	65 (30.1%)	N/A

Values are presented as the mean and standard deviation or as the number and percentage. Visual analogue scale, VAS

### Characteristics of caregivers

The 216 caregivers were all family members of the patients [126 males and 90 females; mean age ± standard deviation (SD): 70.46 ± 9.13 years (range, 20–75)] and included 170 spouse caregivers (78.7%). Most caregivers lived in rural areas (60.6%) and had received a primary school education or less (70.4%). The monthly income of 74.5% of caregivers was less than 500 dollars, and 30.6% of caregivers had cared for the patient for more than 6 months (Table 1).

### Caregiver burden

The average ZBI score for the primary family caregivers was 35.5 ± 7.5. In addition, 15.4% of caregivers presented little or no burden, 60.5% showed moderate burden and 24.1% reported a heavy burden.

### Correlations of caregiver burden with the SSRS and GSE scores

In this study, the relationship among caregiver burden, self-efficacy and social competence was assessed based on the analysis of the correlations among the ZBI, SSRS and GSE scores. The average SSRS and GSE scores of the family caregivers were 43.5 ± 6.4 and 24.6 ± 5.8, respectively. The results demonstrated that a high ZBI score was correlated with low SSRS and GSE scores, suggesting a negative correlation.

### Factors associated with caregiver burden

Univariate analysis showed that the care burden was not related to patients’ age, gender, education level, or tumour site (Table 2), but patients’ degree of pain, primary cancer site, chemotherapy and/or radiation, cognitive dysfunction, functional status and paralysis were associated with the burden of care. Specifically, chemotherapy and/or radiation was positively correlated with caregiver burden.

**Table 2:** Univariate factor analysis of the caregiver burden with patient variables

Variable	Little/no	Moderate	Severe	Difference
Age (years)	71.88 ± 9.02	70.87 ± 8.69	71.34 ± 8.76	*P* > 0.05
Gender				
Female	18 (54.5%)	69 (52.7%)	28 (53.8%)	*P* > 0.05
Male	15 (45.5%)	62 (47.3%)	24 (46.2%)	*P* > 0.05
Education				
≦Primary	22 (66.7%)	88 (67.2%)	35 (67.3%)	*P* > 0.05
≧Secondary	11 (33.3%)	43 (32.8%)	17 (32.7%)	*P* > 0.05
Primary cancer site				
Spine	15 (26.3%)	20 (27.8%)	24 (27.6%)	*P <* 0.05
Other	42 (73.7%)	52 (72.2%)	63 (72.4%)	*P <* 0.05
Chemotherapy and/or radiation for tumour				
Chemotherapy and radiation	10 (27.8%)	15 (23.1%)	28 (24.3%)	*P <* 0.05
Other	26 (72.2%)	50 (76.9%)	87 (75.7%)	*P <* 0.05
Cognitive dysfunction				
Depression	12 (22.2%)	20 (20.6%)	10 (15.4%)	*P <* 0.05
Disruptive behaviour	2 (7.4%)	4 (9.3%)	3 (10.8%)	*P <* 0.05
Functional status (activities of daily living)				
Fully dependent	9 (20.9%)	15 (21.1%)	28 (27.5%)	*P <* 0.05
Other	20 (46.5%)	30 (42.3%)	49 (48.0%)	*P <* 0.05
Tumour site				
Thoracic	16 (48.5%)	64 (48.8%)	25 (48.1%)	*P* > 0.05
Other	17 (51.5%)	67 (51.2%)	27 (51.9%)	*P* > 0.05
Paralysis				
Yes	12 (36.4%)	45 (34.4%)	18 (34.6%)	*P <* 0.05
Pain, VAS score				
*<* 3	7 (21.2%)	28 (21.4%)	11 (21.2%)	*P <* 0.05
≧4	26 (78.8%)	103 (78.6%)	41 (78.8%)	*P <* 0.05

Univariate analysis also revealed that the care burden was not linked to caregivers’ age, gender, education level or relationship with the patient (Table 3), but significant correlations were found between caregivers’ residence, care time and monthly income and the burden of care.

**Table 3:** Univariate factor analysis of the caregiver burden with caregiver variables

Variable	Little/no	Moderate	Severe	Difference
Age (years)	71.01 ± 10.12	70.49 ± 3.68	70.17 ± 8.95	*P* > 0.05
Gender				
Female	14 (42.4%)	54 (41.2%)	22 (42.3%)	*P* > 0.05
Male	19 (57.6%)	77 (58.8%)	30 (57.7%)	*P* > 0.05
Education				
≦Primary	23 (69.7%)	92 (70.2%)	37 (71.2%)	*P* > 0.05
≧Secondary	10 (30.3%)	39 (29.8%)	15 (28.8%)	*P* > 0.05
Place of residence				
Rural	20 (60.6%)	79 (60.3%)	32 (61.5%)	*P <* 0.05
Urban	13 (39.4%)	52 (39.7%)	20 (38.5%)	*P <* 0.05
Relationship				
Spouse	26 (78.8%)	103 (78.6%)	41 (78.8%)	*P* > 0.05
Other	7 (21.2%)	28 (21.4%)	11 (21.2%)	*P* > 0.05
Duration of caregiving				
*<* 6 months	23 (69.7%)	91 (69.5%)	36 (69.2%)	*P <* 0.05
> 6 months	10 (30.3%)	40 (30.5%)	16 (30.8%)	*P <* 0.05
Monthly income				
≦500 US	25 (75.8%)	98 (74.2%)	38 (74.5%)	*P <* 0.05
> 500 US	8 (24.2%)	34 (25.8%)	13 (25.5%)	*P <* 0.05

### Multivariate analysis of the factors associated with caregiver burden

All the variables with a *P* value < 0.10 in the univariate analysis and caregivers’ gender were included in a multiple regression model to control for potential confounders. Multivariate analysis showed that patients’ paralysis, pain score, primary cancer site, chemotherapy and/or radiation, cognitive dysfunction, functional status, GSE score, SSRS score and monthly income were significantly correlated with the burden of care. These clear and key factors explained approximately 59% of the variance in care burden.

## Discussion

This study analysed the factors related to the burden of caring for patients with spinal tumours, thus contributing to the understanding of the care burden experienced by caregivers in Northwest China. This study showed that most of the family caregivers of patients with spinal tumours carried a considerable care burden. The analysis of caregiver burden revealed that the average ZBI score was 35.5 ± 7.5. The factors significantly related to the burden of care included patients’ paralysis, pain score, GSE score, SSRS score and monthly income.

In this study, the sociodemographic survey of caregivers revealed that the family caregivers were typically spouses of the patients with a low education level and an age of approximately 70 years. However, these demographic characteristics did not increase caregiver burden. The patients’ degree of pain was positively correlated with the burden of care. Severe pain affects the quality of life of patients. Pain requires continuous analgesic treatment, which increases the workload and expenditure of caregivers, ultimately increasing the burden of care. Patients’ paralysis was positively correlated with caregiver burden, indicating that patients with spinal tumours needed more supervision and personal care, increasing the burden of care. In this study, most family caregivers earned less than 500 dollars per month. Low monthly income represented a high caregiver burden. Therefore, specific strategies and policies are needed to reduce the burden of care for elderly caregivers and low-income families. For example, the government could increase support for the elderly, increase government financial support, expand medical insurance coverage, and strengthen low-cost and effective nursing services.

As the main resources available for coping with the demands of care, social support of caregivers and caregiver self-efficacy can effectively reduce caregiver burden [18, 19]. This study found that social support was negatively correlated with caregiver burden, which was consistent with the findings of other parallel studies [20, 21]. In China, due to the lack of traditional culture and available services, caregivers usually cannot receive sufficient support from other members of the family. Additionally, social support from other families and professional institutions makes it easier for caregivers to take care of patients and themselves, to cope with stress and, perhaps, to minimize caregiver burden [22, 23]. To reduce caregiver burden, more social support from professional health care institutions and personnel is needed, such as the provision of appropriate information and support by medical staff and the strengthening of family care services for caregivers. Moreover, self-efficacy can reduce caregiver burden [24, 25], lead to more positive emotions and contribute to better physical health [26, 27]. Our previous studies have also confirmed that self-efficacy reduces the burden of care for caregivers and leads to more positive emotions and better physical health [10]. The results of this investigation are consistent with the above results. This investigation showed that self-efficacy is negatively correlated with caregiver burden, indicating that family caregivers with high self-efficacy can better cope with care stress. More attention and support, such as vocational training, should be provided to the family caregivers of spinal tumour patients to help them improve their self-efficacy and thereby reduce the caregiver burden.

There are some limitations to this study. The sample size of this study was relatively small, and only a single spine centre in western China was involved. Considering the cross-sectional nature of this study, we cannot draw conclusions about causal relationships. Therefore, longitudinal studies are recommended to assess the factors related to caregiver burden. Despite these limitations, this study identifies the factors closely related to caregiver burden and proposes improvement strategies and interventions to reduce the burden of caring for patients with spinal tumours.

## Conclusion

Family caregivers of patients with spinal tumours often experience poor quality of life and immense psychological and economic stress. In China, little information is available on the factors related to the burden of caring for patients with spinal tumours. This study provides information on the care burden of family caregivers of patients with spinal tumours.

In this study, we gained insight into the complex relationships of the factors related to caregiver burden. The results indicated that the patients’ paralysis, pain score, primary cancer site, chemotherapy and/or radiation, cognitive dysfunction, functional status, GSE score, SSRS score and monthly income are directly correlated with caregiver burden. In addition, chemotherapy and/or radiation was positively correlated with caregiver burden. This investigation also showed that self-efficacy is negatively correlated with caregiver burden, indicating that family caregivers with high self-efficacy can better cope with care stress. More attention should be paid and support should be provided to the family caregivers of patients with spinal tumours to improve their self-efficacy. These findings may lead to discussion and action, which may help reduce the short-term or medium-term burden on family caregivers for patients with spinal tumours. To better understand the burden of family caregivers for patients with spinal tumours in northwestern China, more in-depth research is needed. We should also conduct a longitudinal cohort study to deepen our understanding of the factors involved in family caregiver care burden and the impact of these factors on caregivers. These measures will ultimately lead to better patient care.

## Data Availability

The datasets supporting the conclusions of this article are included within the article. The raw data can be requested from the corresponding author on reasonable request.

## Abbreviations

GSE: General self-efficacy scale
SD: Standard deviation
SSRS: Social support rating scale
ZBI: Zarit burden interview
